# A preliminary study on automated freshwater algae recognition and classification system

**DOI:** 10.1186/1471-2105-13-S17-S25

**Published:** 2012-12-07

**Authors:** Mogeeb AA Mosleh, Hayat Manssor, Sorayya Malek, Pozi Milow, Aishah Salleh

**Affiliations:** 1 Artificial Intelligent Department, Faculty of Computer Science & Information Technology, University of Malaya, Kuala Lumpur, Malaysia; 2 Institute of Biological Sciences, Faculty of Science, University of Malaya, Kuala Lumpur, Malaysia

## Abstract

**Background:**

Freshwater algae can be used as indicators to monitor freshwater ecosystem condition. Algae react quickly and predictably to a broad range of pollutants. Thus they provide early signals of worsening environment. This study was carried out to develop a computer-based image processing technique to automatically detect, recognize, and identify algae genera from the divisions Bacillariophyta, Chlorophyta and Cyanobacteria in Putrajaya Lake. Literature shows that most automated analyses and identification of algae images were limited to only one type of algae. Automated identification system for tropical freshwater algae is even non-existent and this study is partly to fill this gap.

**Results:**

The development of the automated freshwater algae detection system involved image preprocessing, segmentation, feature extraction and classification by using Artificial neural networks (ANN). Image preprocessing was used to improve contrast and remove noise. Image segmentation using canny edge detection algorithm was then carried out on binary image to detect the algae and its boundaries. Feature extraction process was applied to extract specific feature parameters from algae image to obtain some shape and texture features of selected algae such as shape, area, perimeter, minor and major axes, and finally Fourier spectrum with principal component analysis (PCA) was applied to extract some of algae feature texture. Artificial neural network (ANN) is used to classify algae images based on the extracted features. Feed-forward multilayer perceptron network was initialized with back propagation error algorithm, and trained with extracted database features of algae image samples. System's accuracy rate was obtained by comparing the results between the manual and automated classifying methods. The developed system was able to identify 93 images of selected freshwater algae genera from a total of 100 tested images which yielded accuracy rate of 93%.

**Conclusions:**

This study demonstrated application of automated algae recognition of five genera of freshwater algae. The result indicated that MLP is sufficient, and can be used for classification of freshwater algae. However for future studies, application of support vector machine (SVM) and radial basis function (RBF) should be considered for better classifying as the number of algae species studied increases.

## Background

Algae have been long been used to assess environmental conditions in aquatic habitats throughout the world [1]. Algae respond to wide range of pollutants. They provide an early caution signal of worsening ecological condition. They are highly sensitive to changes in their environment and therefore a good indicator [2]. Shifts in abundance of algal species can be used to detect environmental changes, and also to indicate the trophic status and nutrient problems in lake [3]. Nutrient stimulation of algal growth made algae part of the problem in the eutrophication of lakes, and trophic status of lakes can be monitored by algal taxa found in them.

Algae from the division of Bacillariophyta and Chlorophyta especially the desmids (e.g., *Scenesdesmus*) are highly sensitive to changes in the environmental parameters that could be considered as a bio-indicator for monitoring water quality [4-6]. However, several species of algae are capable to produce potentially harmful toxins as unpleasant taste and odour. Chlorophytes are often abundant in eutrophic lakes. Blooms of *Staurastrum *have created grassy odour problems. *Navicula *is a member of the group of algae called Bacillariophyta. The hard cell walls of *Navicula *do not decompose even when the cells die. The remaining skeletons of the cells create problems when they clog the filters at water treatment plants. Cyanobacteria are known to produce nuisance blooms in eutrophic waters. Furthermore, some species of cyanobacteria contributes to toxin, taste, and odour problem in water. Some types of cyanobacteria such as *Microcystis*, and *Anabaena *are toxin and odour producing. Cyanobacteria has become a critical problem over worldwide because of it is toxicity, and it is widely spread in eutrophic lakes. Surveys studies carried out in different countries demonstrated that about 75% of lake water samples contain toxic cyanobacteria [7,8]. Moreover, cyanobacteria as a control parameter for water quality was included and recommended to be as a factor of risk assessment plans and safety level such as World Health Organization (WHO) and several national authorities worldwide [9-11].

However, identification of algae presents a problem in their taxonomy and the application of the organisms in environmental studies. Several studies reported the conventional identification of algae by using microscopy images is time consuming with the general decline in competent algae taxonomists. This has led many researchers to develop several systems to automate the analysing and classifying algae images [12,13]. An automated computer-based recognition and classification system for rapid identification of microorganisms such as many algae will certainly reduce the burden of routine identifications borne by taxonomist whose service are needed in biodiversity studies [14]. ANN based automated algae recognition is advantageous due to its learning capability from a given dataset, and it does not require a rule base to determine outcome. ANN is also capable to perform mapping arbitrarily between input and outputs. It can also be used in a wide variety of domains for classification, prediction, approximation, and clustering. It is also resistant to noise in the input data. ANN has been successfully applied for classification of two co-occurring species of *Ceratium *by applying the back propagation learning method with three hidden layers [15-17]. ANN has also been used widely to identify different type of algae species of lake water samples, and microorganism. Several researches were extracted a set of suitable features of algae images such as Fourier descriptors, geometrical features, and features characterizing of grey level distribution in a region to use it for training process of ANN [18,19]. Different types of ANN have been employed to classify algae images such as feed-forward multilayer, back propagation error, Radial Basis, and support vector machine. For example, support vector machine (SVM) as a type of ANN had been used together with radial basis function kernel to distinguish between 241 species of marine phytoplankton with 89% accuracy [20]. Research reported that recognition accuracy rate is mainly depends on image segmentation process, selected features to be extracted, and the classifier type or the type of ANN. Research used many segmentation methods for detecting algae objects in microscope images, a large variety of features had been extracted to enhance the recognition process including geometrical feature, colour features, and textures features. Geometrical feature is given measurement parameters about the object shape such as size, length, width, and texture features includes some features about image such as moments varying, image histogram, image texture, and image spectrum [21].

However, most efforts for automated analysis and identification of algae images were limited to some specific type of algae division only. This is because of the difficulties in implementation of an application that can detect all types of algae division due to the variation found in algae shapes, properties, and colours. So far, only a few or limited studies exist on automated identification of tropical freshwater algae [22].

Therefore, this study is an early attempt to devise an automated recognition and classification system for several common algae. A combination of image processing with ANN approaches used to automatic detection and recognition of some selected freshwater algae genera. These algae were from the divisions of Bacillariophyta(*Navicula*), Chlorophyta (*Scenedesmus*) and Cyanobacteria (*Chroococcus*, *Microcystis *and *Oscillatoria*) found in tropical Putrajaya Lake. Although this lake is a mesotrophic lake, there is a need to monitor changes in its water quality as socio-economic developments take place in surrounding areas. Automated recognition and classification system for algae will be one of the several tools to be developed for monitoring algae diversity of and hence, water quality changes, the lake. This study is also an extension of previous studies by other workers who focused on certain algal taxa only.

## Methods

### Study site and data

Putrajaya Lake is a man-made freshwater lake. The lake, which covers an area of 650 ha, is located at the new capital city of Malaysia known as Putrajaya. The lake was constructed to provide a landscape feature and varied recreational activities for the city population as well as creating wildlife habitats [23]. Putrajaya Lake is warm polymictic, oligotrophic to mesotrophic, and is located at the south of the densely inhabited Klang Valley, Malaysia. Major inflows from upstream outside surrounding areas contain certain level of pollutants. Nutrient loading at the lake are mainly come from non-point sources. These include the use of agrochemicals, fertilizer, land clearing, and soil leveling at the surrounding areas. Freshwater algae images used in this work have been captured from water samples collected from different locations at Putrajaya Lake, Malaysia. Water samples were analyzed and examined by using electronic microscope Manufactured by Thermo fisher scientific company model(MTC#B1-220ASA), and freshwater algae images were transferred to digital storage devices by using a Dino-Eye Eyepiece camera Manufactured by Dutech scientific company model (AM423X) which attached to the microscope lens, and connected with personal computer via USB port for image acquisition.

**Figure 1:**
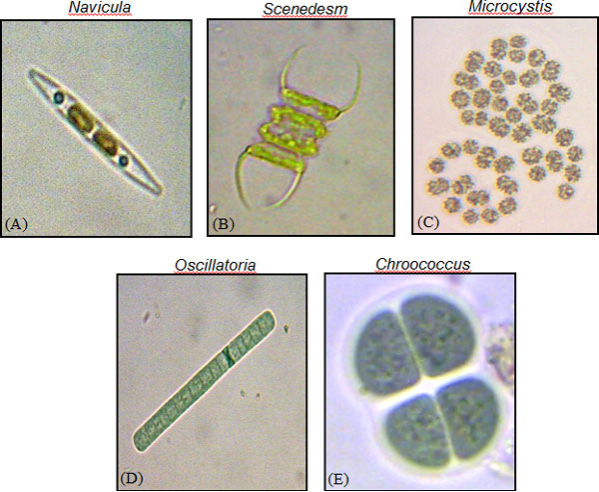
**Samples of algae images used in this study**. Images of algae genera used in this study (a) *Navicula*. (b) *Scenedesmus*. (c) *Microcystis*. (d) *Oscillatoria*. (e) *Chroococcus*.

Image acquisition was performed using attached camera assisted with computer software (DinoCapture 2.0), and captured image resolution was 1280 × 1024 pixels. Manual identification of algae species were carried out based on their taxonomic characteristic by Aishah [24]. The data set included three genera of Cyanobacteria, one genera of Chlorophyta, and one genus of Bacillariophyta as shown in Figure 1. 100 image samples collected to be used for each selected algae genus. The algae image samples are then classified into two groups, training group which contains 40 images for each algae genus, and testing group which contains 60 images for each algae genus. The operating system platform used in this work was Intel CORE i5 CPU, 4 GB RAM, Windows 7 professional (64 bit). Image processing and other related approaches were performed using computer software MATLAB 7.0.

### System development

**Figure 2:**
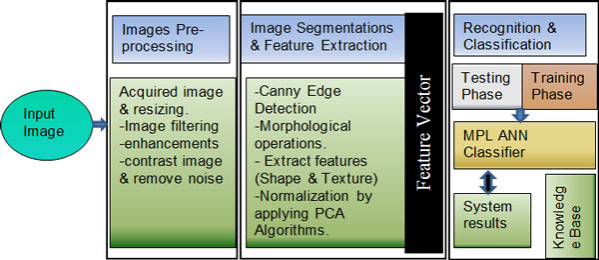
**System flowchart diagram**.

Matlab 7.0 was used to develop the automated freshwater algae detection and classification prototype. Matlab 7.0 has the ability to integrate technical computing environment which is suitable for algorithm design and development. It is considered as a high-level programming language which includes a lot of functions that support image processing and classification methods. The development process of the automated prototype involves image preprocessing, segmentation, feature extraction and classification. The system architecture is shown in Figure 2.

### Image pre-processing

Images captured from the microscope mostly suffer from noise and low contrast quality, it may contain some hole, small objects or unwanted area, and it contains mostly unavoidable scum exist beside the target cells. Image pre -processing was carried out to enhance the captured image features. It removes noise to improve intelligibility and appearances of the images. Basic steps of image preprocessing were used and listed as follows:

1. Captured images are uploaded to the system using graphical user interface (GUI).

2. Contrast enhancement was performed to enhance uploaded images, to remove dark area, to increase image brightness, and to make images clearer. Histogram Equalization is applied to enhance the contrast of the color image intensity, before the image is transferred to gray scale image [25]. The frequency occurrence of the pixel intensities was given by the histogram and mapped to a uniform distribution. This step was performed to improve the appearance of the images in terms of the image contrast.

3. Image converted from gray scale to binary image, and image complements obtained to produce image background in black color and image objects in white color.

4. Median filter (size 3 × 3) was used to reduce image noise, and to preserve edges. Some unwanted area and small objects were removed when the median filter was applied.

### Image segmentation

Image segmentation process was used to isolate the individual objects in captured images. An algae sample usually contains foreign objects including other microorganisms. Image segmentation was used to identify the number of detected object in binary image. Image segmentation uses the binary images which had been pre- processed previously. In this study, we used Canny edge detector algorithm to perform image segmentation which is a powerful edge detector for image segmentation [26]. It was used to identify discontinuities in an image intensity value or the edge of the image. The steps are as described as follows:

a) Gaussian filter was applied to smooth the image. It was used with a specified standard deviation, σ, to reduce noise.

b) The local gradient (1), and edge direction (2), were computed at each point. The Gx and Gy were calculated by first derivative of the intensity pixels. An edge point is identified to be a point of locally maximum in the direction of the gradient.

(1) $$g\left(x,\;y\right)={\left[G_{x}^{2}+G_{y}^{2}\right]}^{1/2}$$

(2) $$\alpha \left(x,\;y\right)=tan^{-1}\left(G_{x}/G_{y}\right)$$

c) Then the non-maximal suppression in the gradient magnitude image was used to give a thin line, which was the ridge of the edge points determined in (2). The ridge pixels were then threshold.

d) Finally, the algorithm performed edge linking by incorporating the weak pixels that were connected to the strong pixels.

Then, essential morphological operations performed on binary images such as image border removal, filling of boundary area, and exclusion of any small region that are < 50 pixels. Morphology operation is a set of image processing operations that process images based on shapes. In our system, we used dilation and erosion which considered the most basic morphological operations. To overcome with the problem of objects overlapping, each object was counted as a single item by the image analysis process, therefore it was necessary to separate individual objects. Regions with a maximum length of the rectangle fully enclosing the region > 50 pixels in length and perpendicular of at least 50 pixels were copied to a new binary image. These regions typically represented overlapping objects and the process resulted in their separation from isolated objects and from other regions.

**Figure 3:**
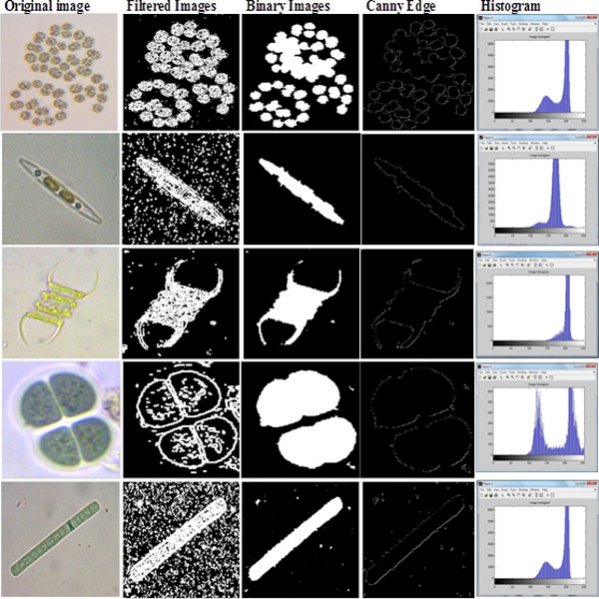
**Image pre-processing and segmentation process**.

Image segmentation is used to separate the input images into multiple images based on the number of detected objects, where each image contains one object only and each sub images would be processed individually. The region of binary image was detected using Canny edge approach, and each region is represented on individual sub image. Each sub image was used as a mask to obtain the same region of original image (colour image); both regions of colour and binary images was associated with corresponding index number to extract features of colour and binary image for the same image and store it in database file. Image pre-processing, image segmentation, and morphological operation methods are associated with some image samples as shown in Figure 3, and Additional File 1.

### Feature extraction

**Figure 4:**
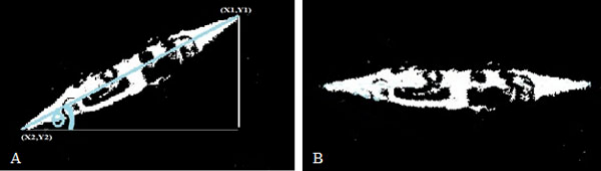
**Angle of inclination with object auto alignment function**. (a) Auto-Alignment process step. (b) Image after Alignment Process.

Feature extraction used to transform binary and colour image from the pre-processed stage into a set of parameters that described the algae features. Feature extracted from the pre-processed algae image using both binary and colour image include: shape, area, minor and major axis, perimeter and Fourier spectrum with principal component analysis (PCA). The details of each extracted feature are described as follows:

**Shape feature: **one of our novel methods was proposed to develop a simple shape classifier and applied it before shape extraction method is performed, a simple classification function that differentiates between the algae shape was created to categorize common algae shapes into three categories. The simple classification function was used to detect the three categories of input algae images. The function identified the algae shape to equal to '0' if circular, '1' for spiral, and '-1' if irregular. This function was used to improve the accuracy rate, and to optimize the time of image recognition process [27]. Results obtained from shape extraction will be included as one of the input parameters to algae classification neural network. A simple function was developed to obtain the angle of inclination for image objects automatically, and then the angle is used to rotate the algae image to be aligned horizontally as shown in Figure 4. This routine is calculated the angle of inclination automatically by obtaining the longest path that existed between each two point on algae boundary. Three identified points P1 (X1, Y1), P2(X2, Y2), and point of origin P(0,0) were used to find the angle of inclination by using the following equation:

(3) $$\theta =tan^{-1}\left(m1-m2\right)/\left(1+m1*m2\right)$$

Where m1 and m2 is the slope of lines that form the angle which were obtained by using the following equation:

(4) $$m1=\left(Y2-Y1\right)/\left(X2-X1\right)$$

(5) $$m2=\left(Y1-Y0\right)/\left(X1-X0\right)$$

This routine is designed to align the rotated shape into horizontal lines which ease the feature extraction process, and also improve the accuracy and performance of recognition process.

**Figure 5:**
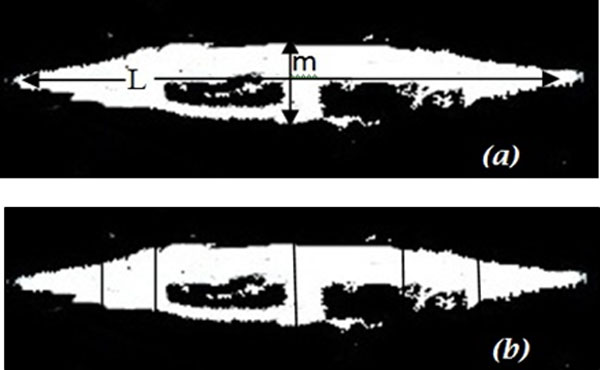
**Minor, Major, and object width feature extraction**. (a) Major and minor axes measurement. (b) Object width measurements.

**Major and minor axis feature: **Major and minor axis of an image are extracted where two points were identified automatically by calculating the maximum distance between given points in objects vector as mention previously. The major axis represents the line segment connecting between the base points in X axis, and minor axis represents the maximum width which is perpendicular to the major Axis as shown in Figure 5(a). Actually, the major and minor axis is represented the length and width of algae objects.

**Object width factor: **to differentiate between similar algae in shape the object width factor was calculated by slicing across the major axis and parallel to minor axis, then feature points were normalized into a number of vertical strips, and for each strip the ratio of strip length to the object width was calculated as the following equation.

(6) $$R_{c}=W_{c}/L$$

Where R_c _is the ratio at column c, W_c _is the width of object at column c, and L is representing the object length as shown in Figure 5(b). Object width factor results then normalized to obtain five features only.

**Figure 6:**
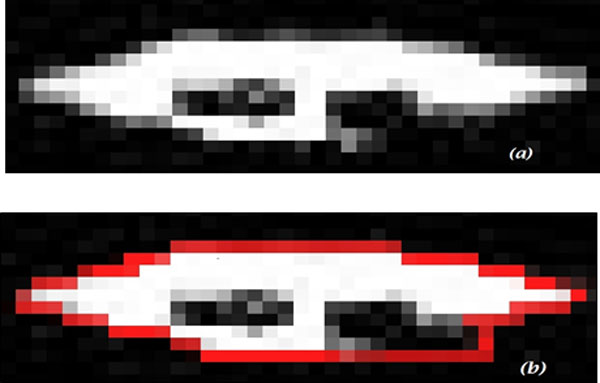
**Perimeter and area feature extraction**. (a) Extract Perimeter. (b) Area extraction.

**Area: **The area is represented by the actual number of white pixels in the selected object region. The object area was calculated by counting the number of white or '1' pixels inside the object boundary as shown in Figure 6(a). The area was included in this study as one of the feature parameters used for classifying process.

**Perimeter: **The perimeter of object was the summation of the distance between each adjoining pair of pixels around the object border; it is shown in red pixel in Figure 6(b). It included in our features because it gives an indication about the image object size.

**Fourier spectrum with PCA feature extraction: **Fourier spectrum was applied to extract some texture feature for increasing the accuracy of the image detection. Fourier spectrum is ideally suitable for describing the directionality of periodic or almost periodic two-dimensional patterns. The spectrum features are expressed in polar coordinates to yield a function *S *(r, *θ*). Radius function (*P_1 _*(*r*)) and angle function (*P_2 _*(ø) obtained by annularity sampling of the function *S *(r, *θ*) are one-dimension functions. Radius function, (*P_1 _*(*r*)), reveals energy distribution information with different frequency.

Feature sets extracted from an algae object may contain some redundant feature. PCA approach is used widely in most image processing application to reduce the number of features by normalization process. It has de-correlation ability that serves to de-correlate redundant features, and its energy packing property serves to compact useful information into a few dominant features [28]. The PCA algorithm is also used to reduce and summarize the extracted features of the Fourier Spectrum method by removing redundancies. Eight Eigen value extracted and included in our feature extraction process.

### MLP ANN for classification

Multilayer perceptron network (MLP) trained with back propagation error algorithm ANN was used to perform classification on extracted feature vectors [29]. These types of ANN are widely used for pattern recognition and classification. In this study one hidden layer feed forward neural network was chosen mainly because it has been proven that such a topology can approximate any continuous function [30-32]. Devilliers and Barnard [33] found that the use of two hidden layers was only justified for the most esoteric applications. The hyperbolic tangent transfer function was used as recommended by most of researchers. The ANN architecture consists of three layers, the input layer which has 21 input nodes - hidden layer include 8 nodes and output layer include 5 nodes. The standard root mean squared error function (RMSE) was used to assess network performance, and a momentum value of 0.05 was set based on trial and error. With the above parameters fixed, optimal step sizes taken in weight space were a function of the learning rate of 0.05 with an epoch size of 400.

The input to ANN is a vector feature dimension that includes 21 features which extracted from input image. R represents the number of features in the input vector, Q is the total number of training (inputs, outputs) pairs as shown in Additional File 2. The element of vector features is shown in Table 1.

**Table 1:** Extracted feature of algae used in this study.

**Feature No**.	Feature Descriptions
**F1, F2, F3**	Shape index, Major Axis, Minor Axis
**F4, F5**	Area, Perimeter
**F6, F7, F8**	Minor/Major, Area/Major, Perimeter/Major
**F9-F13**	Object Width Factor Strips
**F14-F21**	Fourier Spectrums Normalized by PCA

During the training phase the input data and desired responses were fed into the network. The network uses momentum learning algorithm to determine the weights in the network and after each presentation the weights were adjusted to minimize the error between desired and actual output. As training progressed the error between the desired response and the network output dropped towards zero. As an MLP with hidden layers could be approximate virtually any input-output map, it was possible that a network could have been over-trained, i.e. a network that classified the training data perfectly but unable to generalize and classify new 'unseen' data. To improve generalization, 10% of the input data was set aside for cross validation. The training was stopped when the error in the cross validation dataset began to increase. Testing dataset was then used to avoid biasness in result. This was a set of images that are not used for training the ANN.

## Results

**Figure 7:**
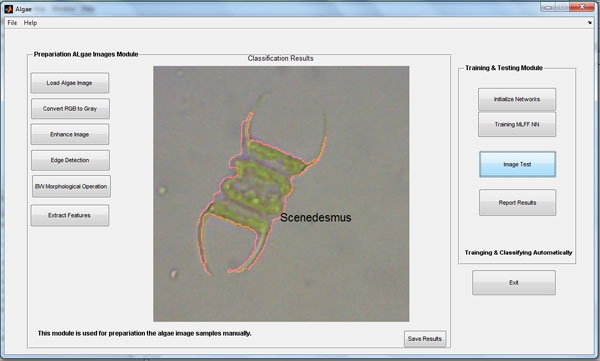
**System GUI example for classification results**. (Testing mode).

The automated algae recognition system graphical user interface (GUI) is shown in Additional File 3 &4, and in Figure 7. The system interface allows user to perform image preprocessing and classification automatically in simple and easy steps.

**Figure 8:**
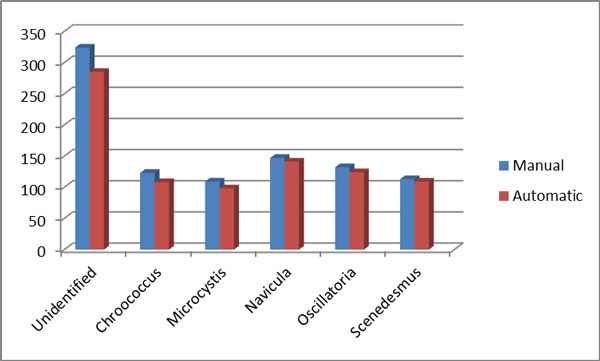
**Comparison results between manual and automatic process**.

**Figure 9:**
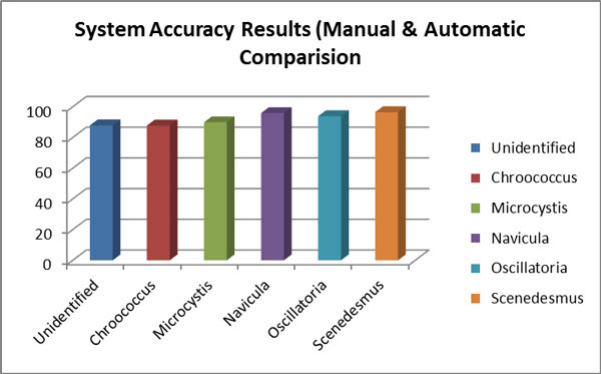
**Accuracy results for manual and automatic Methods**.

In order to test the accuracy of the system testing was carried out for a total of 50 testing images that has not been used for the training of MLP. Sixty images of each genus of selected fresh water algae were used in this study. Two test methods are used to evaluate system accuracy and performance which are the testing system functionality method and the comparison method for inter and intra results of both manual and automatic recognition. Table 2 shows the comparison result between the manual and computer-based classification of selected algae from the available image dataset samples. The number of extracted region from image samples for each alga was examined in both method manual and automatic approach to distingue between alga and other objects as shown in graph chart on Figure 8. The process for separating the objects found in the image resulted in some short irregularly shaped image regions containing the algae. The MLP was more like to misclassify these small segments, and the other small object was excluded during the segmentation process. The foreign objects found in the algae images were classified by the MLP as unidentified. Based on the comparison results of inter image test the algae identified by the automated system were within 90% of the manual classification of the region as shown in Figure 9. The results of comparison showed that the automated system was able to identify the algae in given images within the approximate accuracy of manual procedure.

**Table 2:** Comparison results between manual and automated classification process for testing dataset.

Detected Object	Manual	Automatic	Similarity %
Unidentified	324	285	87.9%
*Chroococcus*	123	108	87.8%
*Microcystis*	109	98	89.9%
*Navicula*	147	141	95.9%
*Oscillatoria*	132	124	93.9%
*Scenedesmus*	113	109	96.5%

**Figure 10:**
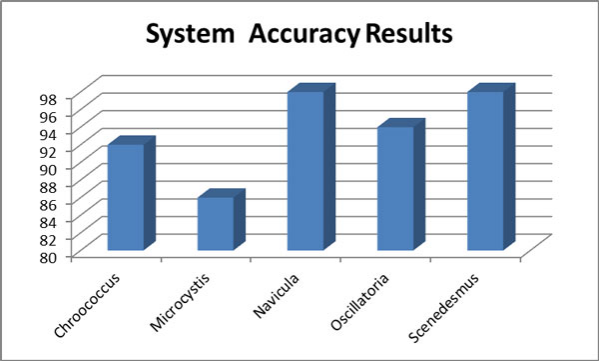
**System recognition accuracy results**.

The proposed system evaluated to measure the accuracy of classifying process between image data set which considered intra testing comparison. The actual classification accuracy resulted by the use of confusion matrix as shown in Table 3, and illustrated in Additional File 5. In this matrix classification result is given by the comparison between the automated analysis with the desired classification (as defined by a human expert). The results demonstrated that the system identified most of input algae images successfully with 93% overall accuracy. The average recognition accuracy of the system 93% and is shown in Figure 10.

**Table 3:** Confusion matrix for testing dataset

Name	No. of Test samples	System recognition results					Unknown	System Accuracy
				
		**Chr**.	**Mic**.	**Nav**.	**Osc**.	**Sce**.		
***Chroococcus***	50	46	2	0	0	0	2	92%
***Microcystis***	50	2	43	0	0	0	5	86%
***Navicula***	50	0	0	49	1	0	0	89%
***Oscillatoria***	50	0	0	2	47	0	1	94%
***Scenedesmus***	50	0	0	0	0	49	1	98%

## Discussion

In this study, we selected specific fresh water algae which impacted strongly the water quality. For example, in different studies performed in Malaysia to asses eutrophication status for 90 lakes, they reported that 56 lakes or 62% were eutrophic or in bad situation which requires immediate rehabilitation and restoration, also they found that the other 34 lakes which represent 38% of the study is classified as mesotrophic [34-36].

The main objective for this study was to develop a computer system to identify, and classify some types of algae. The system is designed and implemented in Matlab environments with friendly interfaces that make it easier for users. System accuracy and performance were calculated by comparing the automated and manual comparison for testing datasets, and by calculating the time of training and recognition process. The automated procedure for training process takes approximately 5 minutes; and the time required for identifying and classifying of input images is varying between 1 to about 1.5 minute. The comparison between the manual and automatic classification of each object found on a particular image which has been identified and extracted resulted in discarding of the of the overlapping images. The highest accuracy rate was achieved for identification of *Scenedesmus *as this alga has the most distinct feature compared to the other algae genus used in this study. Meanwhile *Chroococcus *has the lowest classification rate because of the process for separation resulted in the production of some short, irregularly shaped image region representing the algae. *Microcystis *which is circular in shape is difficult to distinguish because these algae exist in colonies and the images captured are prone to overlapping which cause the MLP to misclassify the algae to unidentified. The accuracy rate for *Navicula *and *Oscillatoria *can be misclassified with each other by automated system as their spiral shape seems similar for the classifier and extracted feature for both of them matching in some parameters. MLP was used in this study instead of SVM or RBF because the data utilized in this study are limited to small number of algae and also limited numbers of extracted features were used. The limited number of feature has been utilized in this study because of the selected features are sufficient to detect and classify selected algae used in this study with considerably high accuracy rate. Furthermore, MPL performs faster as compared to the other types of ANN when data volume is not an issue as the number of algae increases with the number of extracted features SVM and RBF are more suitable option. The overall system accuracy of developed system depends essentially on the ability of system to detect object within input image and the ability of the classification system to identify the detected object based on the extracted feature. Accuracy rate achieved in this study is acceptable and consider higher rate if compared with other similar studies. The system is developed essentially to support the process of monitoring water quality by detection some selected freshwater algae in Putrajaya Lake. Results showed that system able to achieve such tasks by providing the necessary data about the density and gens of selected algae.

## Conclusions

In this paper, we presented an image processing techniques with ANN approach to identify and classify selected genus of freshwater algae from three different divisions of fresh water algae which varies in sizes and shapes. This study illustrated that computational recognition approach is important for freshwater algae, and prove that the classifying process is feasible for automatic identification of the selected freshwater algae. The better accuracy resulted was obtained due to the well preprocessing used techniques, and also due to the specific features selected during extract feature process. In addition, system reliability was dependent more on the combination of approaches used for image pre-processing, segmentation approach used, well selected features, and the training of data set. Testing results also showed that developed system was reliable to be used for monitoring water quality of Putrajaya Lake. The main limitation of our system its inability to work well with images that include a huge number of objects. We would like to solve these limitations in our future work and make the system even more robust in future studies.

## Competing interests

The authors declare that they have no competing interests.

## Authors' contributions

SM headed the study and structured the whole research. MAAM and HM assisted in model development and manuscript writing. PM and AS assisted in manuscript writing. All authors contributed in this study.

## Supplementary Material

Additional file 1 **Example for morphological operation steps**. Click here for file

Additional file 2 **MPL ANN architecture**. Click here for file

Additional file 3 **System GUI example for pre-processing in (preparation mode)**. Click here for file

Additional file 4 **System GUI example for edge detection in (preparing mode)**. Click here for file

Additional file 5 **Confusion matrix chart for data test images**. Click here for file
