# Supplementary figures and images for: A preliminary study on automated freshwater algae recognition and classification system
Source: BMC Bioinformatics. 2012 Dec 7;13(Suppl 17):S25. doi: 10.1186/1471-2105-13-S17-S25 (PMC3521397; doi:10.1186/1471-2105-13-S17-S25)

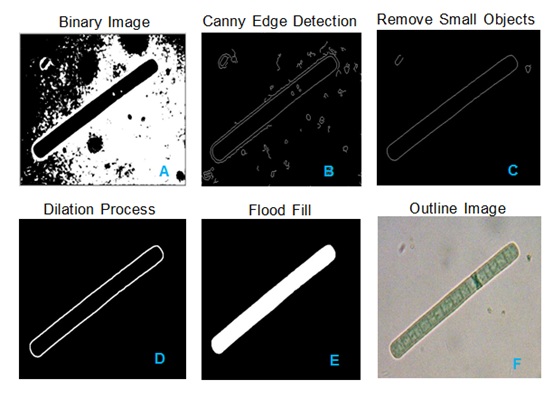

Supplement: Additional file 1 — Example for morphological operation steps. [file 1471-2105-13-S17-S25-S1.jpg]

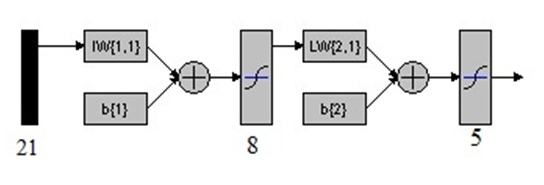

Supplement: Additional file 2 — MPL ANN architecture. [file 1471-2105-13-S17-S25-S2.jpg]

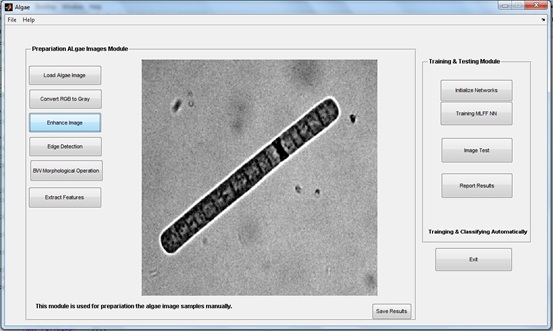

Supplement: Additional file 3 — System GUI example for pre-processing in (preparation mode). [file 1471-2105-13-S17-S25-S3.jpg]

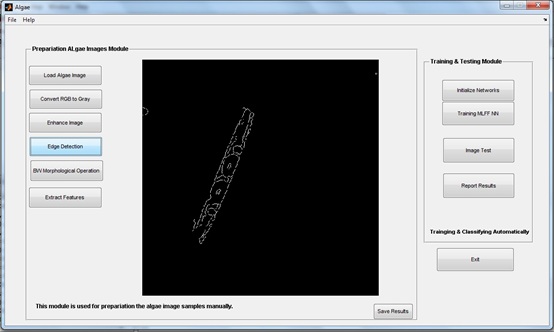

Supplement: Additional file 4 — System GUI example for edge detection in (preparing mode). [file 1471-2105-13-S17-S25-S4.jpg]

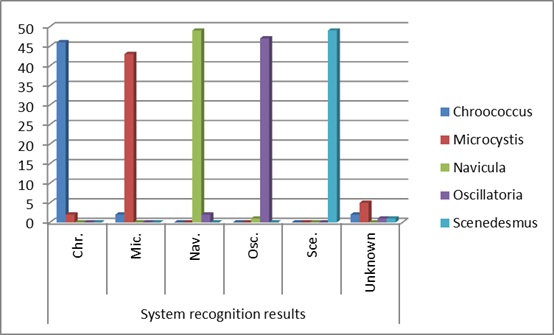

Supplement: Additional file 5 — Confusion matrix chart for data test images. [file 1471-2105-13-S17-S25-S5.jpg]
